# Complete Genome Sequence of *Kaistia* sp. Strain 32K, Isolated from Soil as a Mixed Single Colony with *Methylobacterium* sp. Strain ME121

**DOI:** 10.1128/MRA.00019-21

**Published:** 2021-03-11

**Authors:** Masahiro Ito, Tetsu Shimizu, Akira Nakamura

**Affiliations:** a Graduate School of Life Sciences, Toyo University, Itakura-machi, Gunma, Japan; b Bio Resilience Research Center, Toyo University, Itakura-machi, Gunma, Japan; c Faculty of Life and Environmental Sciences, University of Tsukuba, Tsukuba, Ibaraki, Japan; d Microbiology Research Center for Sustainability (MiCS), University of Tsukuba, Tsukuba, Ibaraki, Japan; Portland State University

## Abstract

*Kaistia* sp. strain 32K, an aerobic Gram-negative bacterium, was isolated from soil in Japan. Here, we report the complete genome sequence of this bacterium, which has a 5.4-Mbp genome sequence, containing 4,919 protein-coding sequences.

## ANNOUNCEMENT

We report the whole-genome sequence of *Kaistia* sp. strain 32K, which was collected as a soil sample from Tsukuba City, Japan (36.09N, 140.09E). Strain 32K and *Methylobacterium* sp. strain ME121 were isolated from the same soil sample (1). Metabolites produced by 32K promote biofilm formation in coculture with ME121 and accelerate the swimming speed of ME121 (2, 3).

Strain 32K was isolated from a single colony grown in liquid mineral (LM) medium (10.0 g of tryptone, 5.0 g of yeast extract, and 1.0 g of d-mannitol per liter) at 30°C for 18 h (2), and genomic DNA was prepared using the Qiagen Genomic-tip 20/G kit (Japan), according to the manufacturer’s instructions. For long-read sequencing, a DNA library with barcodes added to a sample using the barcoding expansion kit (EXP-NBD104; Oxford Nanopore Technologies [ONT], UK) was prepared using a ligation sequencing kit (SQK-LSK109, ONT) and sequenced with a GridION X5 system (ONT) on an R9.4.1 flow cell (FLO-MIN106; ONT). The raw sequence data were base called using Guppy ver. 4.0.11+f1071ce (ONT); adapter sequences and low-quality data (read length, <10 bp; Phred quality score, <9) were filtered and trimmed using Cutadapt ver. 1.11 (5). The adapter sequences were removed using Porechop ver. 0.2.3, and reads of 1,000 bases or less were removed using Filtlong ver. 0.2.0, yielding 197,268 high-quality paired-end reads. The average length of the long reads was 8,022 bp, and the total number of bases was 1,732,779,920 bp. For short-read sequencing, a DNA library was prepared using the MGIEasy FS DNA library prep set (MGI Tech, China) according to the manufacturer’s instructions, and the library’s quality was confirmed using Fragment Analyzer and the double-stranded DNA (dsDNA) 915 reagent kit (Advanced Analytical Technologies, Inc., USA). Circularized DNA was prepared according to the manual using the prepared library and the MGI Easy Circularization kit (MGI Tech). DNBSEQ 2 × 200-bp paired-end sequencing was performed using the DNBSEQ-G400 sequencing instrument (MGI Tech) according to the manufacturer’s instructions. Adapter sequences were removed using Cutadapt ver. 2.7. About 3.5 million read pairs (1.05 Gbp) were sampled from the sequence from which the adapter sequences were removed using Seqkit ver. 0.11.0, and Sickle ver. 1.33 was used to remove bases having a quality value of less than 20; reads shorter than 127 bases and their paired reads were discarded, yielding 6,002,878 high-quality paired-end reads. The average length of the short reads was 200 bp, and the total number of bases was 1,685,436,000 bp.

High-quality short-read and long-read sequence data were assembled under the default conditions of Unicycler ver. 0.4.7, and the genome was circularized. The results of the coding graph, assembled using Bandage ver. 0.8.1, were confirmed, and the integrity of the assembled genomic data was confirmed using CheckM ver. 1.1.2. The coverage calculated from the total bases used for assembly was 639×. The final chromosome sequence was 5,352,285 bp long (G+C content, 66.5%). The chromosome sequence was annotated using Prokka ver. 1.13 (Illumina, USA), which predicted 4,919 coding sequences, as well as 12 rRNA and 60 tRNA genes.

### Data availability.

The GenBank/DDBJ accession number for the whole-genome sequence of *Kaistia* sp. strain 32K is AP024269. The raw sequencing data were deposited in the DDBJ Sequence Read Archive under accession number DRA011233 (NCBI BioProject accession number PRJDB10901 and BioSample accession number SAMD00262792).
